# Gut Microbiota Characterization in Patients with Asymptomatic Hyperuricemia: probiotics increased

**DOI:** 10.1080/21655979.2021.1976897

**Published:** 2021-09-30

**Authors:** Hai-Tao Yang, Wen-Juan Xiu, Jing-Kun Liu, Yi Yang, Xian-Geng Hou, Ying-Ying Zheng, Ting-Ting Wu, Chen-Xin Wu, Xiang Xie

**Affiliations:** a Department of Cardiology, First Affiliated Hospital of Xinjiang Medical University, Urumqi, China; b Department of Oncology, First Affiliated Hospital of Xinjiang Medical University, Urumqi, China; c Department of Cardiology, First Affiliated Hospital of Zhengzhou University, Key Laboratory of Cardiac Injury and Repair of Henan Province, Zhengzhou, China

**Keywords:** Asymptomatic hyperuricemia, diagnostic model, gut microbiota, probiotics, 16s rRNA gene

## Abstract

Asymptomatic hyperuricemia (AH) is an early stage of gout. Emerging evidence shows that the intestinal microbiota is related to gout. However, the relationship between AH and the intestinal microbiota is poorly understood. Therefore, the aim of the current study was to explore the possible correlation between AH and intestinal flora. We compared the intestinal microbial communities of AH (45 cases) and healthy subjects (45 cases) by 16S rRNA gene sequencing and clustering analysis on the incorporated population. Intestinal-type clustering can be divided into two groups, and significant differences in the proportion of AH are found among different bowel types. Alpha diversity indices were higher in the AH group than in the control group, and beta diversity indices also showed significant differences. A total of 19 genera were found different between the AH group and the control group. Compared with the control group, some probiotics are increased in the AH population. Two groups were ranked by importance of bacteria. We found the different bacteria partially coincided with the important bacteria, and the joint diagnosis level of the important bacteria was good. Conclusion: There were significant differences in the composition of intestinal biota between AH patients and healthy subjects. Some probiotics increased in AH.

## 1. Introduction

With the improvement of people’s living standards and the change in the structure of dietary, the prevalence of hyperuricemia (HUA) has increased year by year and has become a common disease that seriously threatens human health [1]. HUA is a metabolic disease caused by the excessive production of uric acid due to the disorder of purine metabolism and/or the decrease in UA excretion. It is characterized by increasing levels of uric acid in the blood, which is the main cause of gout. The clinical symptoms of HUA can manifest as inflammatory arthritis, pain, and disability. Asymptomatic hyperuricemia (AH) refers to the following disease: the serum uric acid level is elevated, while there are no gout-related clinical symptoms (including arthritis, tophus or uric acid nephropathy, urinary tract stones, etc.), which are in the early clinical stage of gout. In contrast to the HUA, the AH is insidious. Continuous research has shown that continuously increasing UA levels are also closely related to many diseases, such as diabetes [2], hypertension [3], stroke [4], and myocardial infarction [5]. It also suggests the great harm of the AH. Currently, there are no clinical guidelines for when and where patients with AH should be treated for uric acid lowering, and researchers have not yet reached a consensus. Therefore, a study of the further analysis on mechanism of AH is crucial.

Among many causes, the patients’ diets are closely related to the occurrence and development of HUA. In recent years, research has reported that the intake of animal-derived food and legumes is positively correlated with the occurrence of HUA. In this way, controlling the intake of animal-derived foods and legumes is conducive to controlling the risk of HUA [6]. Similarly, controlling the diet of patients with diagnosed HUA can help reduce serum uric acid levels [7]. The long-term stable eating habits of the host will form a specific intestinal flora structure, and different microorganisms in the intestine will produce different functional effects. Not only is the intestinal flora closely related to the health of the host, but the metabolites of the intestinal flora also play an important role. For example, common dietary components can be metabolized by the gut microbiota and produce metabolites that regulate host metabolism (such as dietary choline and trimethylamine). Some of these metabolites can have beneficial or harmful effects on the host [8]. Therefore, the study of the intestinal flora in patients with AH is a significant breakthrough in the study of disease pathogenesis.

In the intestinal flora, the study of probiotics is very important for the occurrence and progression of diseases. Based on the harsh culture conditions of intestinal flora and difficult experimental verification, there are few reports on probiotics of AH, and few studies have systematically reported the characteristics of the intestinal flora of AH. Therefore, this study is the first to use bioinformatics to preliminarily speculate the role of microorganisms through grouping and comparison of AH group and control groups to explore the composition structure of intestinal flora in patients with AH; which bacteria are probiotics for patients with AH, and which bacteria play an important role in the occurrence and development of diseases. It can provide a basis for the study of probiotics and new insights for researchers interested in probiotics in the treatment of AH and gout.

## 2. Methods

### 2.1 Study design and population

This study included 45 patients with AH in the First Affiliated Hospital of Xinjiang Medical University and 45 cases in the control group. The diagnostic criteria for the AH population are as follows: under the condition of a normal purine diet, in two fasting blood uric acid determinations on separate days, if the index of the blood uric acid was more than 360 μmol/L (for woman), or more than 420 μmol/L (for man), it would be diagnosed as HUA; if there was no related complication or gout, it would be diagnosed as AH [9]. If at least one main coronary artery stenosis was larger than 50%, the patient was diagnosed with coronary heart disease, and there were a total of 55 cases of coronary heart disease. According to Hypertension Guidelines in China, 44 patients were diagnosed with hypertension. Patients with diabetes were diagnosed according to China’s Guidelines for the Prevention and Treatment of Type 2 Diabetes Mellitus, and there were 13 diabetes patients. The clinical data of patients were collected, including basic information and laboratory data of the enrolled population. The following patients were excluded to the analysis: 1. Patients diagnosed with heart failure, structural heart disease, and pulmonary heart disease 2. Patients with a history of using antibiotics or probiotics within 3 months. 3. Patients with severe liver and kidney dysfunction, such as patients whose creatinines were not less than 2-fold of the normal upper limit, patients whose aspartate transaminases or alanine transaminases were not less than 3-fold of the normal upper limit, etc. 4. Patients with abnormal stool morphology, such as diarrhea and dry stools. The study design complied with the Declaration of Helsinki, and it was approved by the Ethics Committee of the First Affiliated Hospital of Xinjiang Medical University. Before recruitment, informed consent was obtained from eligible patients.

### 2.2 Past healthy history and clinical data

The past healthy history and clinical data are also collected. For alcohol intake, subjects were classified as heavy drinkers as who drank more than eight standard drinks per week [10]. Our study adopted the World Health Organization definition to define smoker. Those who had smoked continuously or cumulatively for 6 months or more were defined as smokers (current smoking status). Smokers had no longer smoked at the time of the survey and persisted for more than 6 months to become Past Smoking status. Five milliliters of peripheral venous blood was taken from each patient after 12 hours of fasting. The testing laboratory data included routine blood testing parameters, blood biochemical analysis results, renal function parameters and liver function parameters, and blood lipid analysis results.

### 2.3 Fecal specimen collection, DNA extraction, and sequencing

Each participant was provided with a fecal sampler for sample collection. A formal training was provided to all the participants included in study on on how to collect the sample before recruitment. Stool samples freshly collected from each participant were divided into five aliquots of 200 mg, immediately transported to the laboratory and frozen at −80°C. The bead-beating method was utilized to isolate bacterial DNA from fecal samples as described previously [11]. Polymerase chain reaction was applied to amplify the V3-V4 region of 16S rRNA genes by applying the extracted DNA from each sample as the template. All DNA extraction and sequencing were performed by Shanghai Personal Biotechnology Co., Ltd. The sequencing data were processed by adopting the Quantitative Insights Into Microbial Ecology (v1.8.0) pipeline, as described previously [12].

### 2.4 Microbiome data analysis

Cluster analysis based on bacterial species composition was performed to classify the subjects into enterotypes [13]. When exploring the microbial diversity of both groups, we applied dimensionality reduction in bioinformatics. Alpha-diversity was used to assess the richness and diversity of the sample and evaluated by indices such as Shannon, Chao, Ace and Goods coverage. For the analysis of beta diversity, the overall phylogenetic distance between communities was estimated and visualized by utilizing Bray-Curtis distance-based principal coordinates analysis [14]. For the analysis of species differences, we employed linear discriminant analysis effect size analysis, combining nonparametric Kruskal–Wallis and Wilcoxon rank sum tests, with linear discriminant analysis effect quantities [14]. For the linear discriminant analysis effect size, the Galaxy Online Analysis Platform was applied .

### 2.5 Statistical analysis

SPSS version 22 (SPSS Inc., Chicago, IL, USA) and R version 3.2.4 (R Foundation for Statistical Computing, Vienna, Austria) were used for the statistical analysis of clinical data. Continuous variables were analyzed using Student’s t-test. The chi-square test or Fisher’s exact test was utilized to analyze categorical variables. P-value were calculated for all analysis and when it was less than 0.05 [15], results were considered statistically significant. To identify biomarkers, a random forest analysis method was used. Subsequently, we used receiver operating characteristic (ROC) curve analysis to evaluate the accuracy of each variable in predicting AH. To rank the variables according to their predictive accuracy, we used the area under the curve (AUC) values as described previously [11].

## 3. Results

To explore a possible correlation between gut microbiota and AH, we compared the gut microbiota communities of AH patients with those of healthy subjects via 16S rRNA gene sequencing and found significant compositional differencesof gut microbiota in AH patients compared with healthy subjects. Probiotics increased in AH group. These results suggest that there are dynamic changes in gut microbiota in the development of disease, and these dynamically changed bacteria may play an important role.

### 3.1 Basic population information and intestinal microbial composition

**Figure 1.**
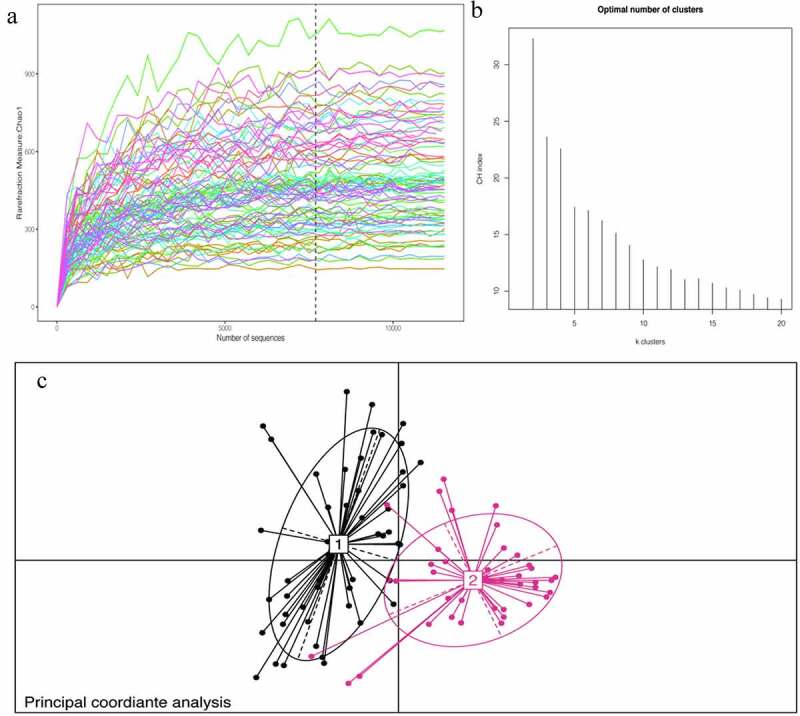
A:Chao1 curves of each sample; the estimated operational taxonomic unit richness basically approached saturation in all samples.B:The number of enterotype was calculated according to CH index. C: Jensen–Shannon-based principal coordinates analysis

**Figure 2.**
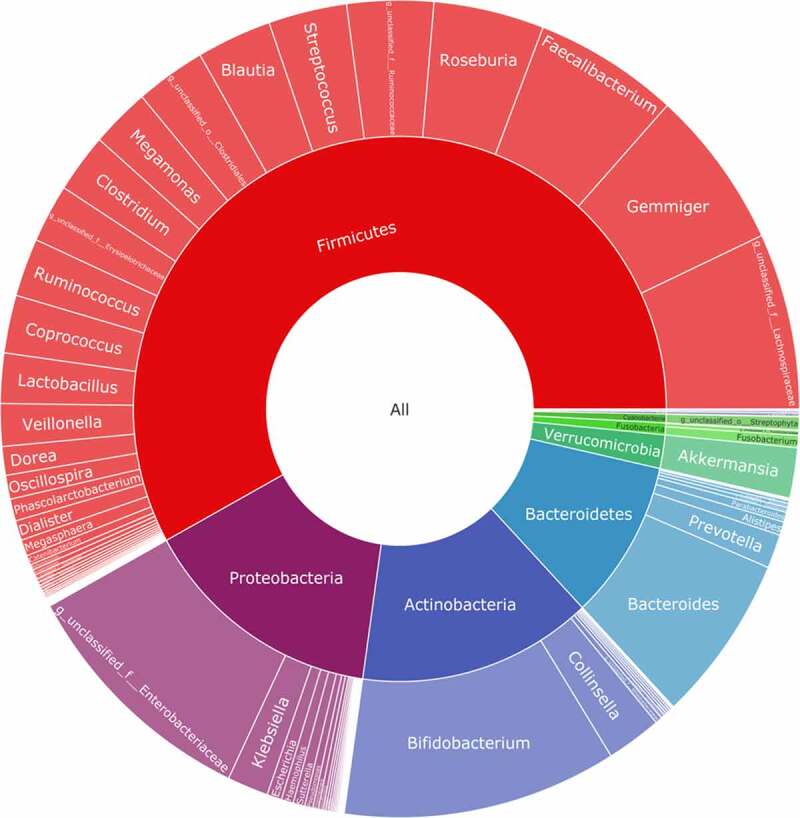
Taxonomic composition of the gut microbiome of patients. double pie chart (inner circle: phylum level, outer circle: genus level)

The baseline characteristics of the patients are shown in Table 1. The average age of the selected patients was 60 years old. Patients with AH account for 50% of the total population; patients with coronary heart disease account for 60.11% of the total population; patients with hypertension account for 48.89% of the total population; and the diabetes population accounts for 12.22% of the total population. The percentage of smokers among the included patients was 35.56%, and 28.88% patients are alcohol users. We recorded the biochemical examination index at admission and conducted 16S rRNA sequencing on 90 patients’ stools. To determine whether the sample size was sufficient and to estimate the community richness, a species accumulation curve was applied in this study (see Figure 1(a)). From the curve, it could be seen that all were stable the curve reach the saturation, indicating that the included sample size was sufficient to cover the community richness for statistical analysis. To display the microbial species composition of the enrolled sample population, a double-layer pie chart was used, as shown in Figure 2. At the bacterial phylum level, the relative abundances of *Firmicutes, Actinobacteria, Bacteroidetes*, and *Proteobacteria* were 58.01%, 14.24%, 9.48%, and 14.77%, respectively; at the genus level, the relative abundances of *Faecalibacterium, Gemmiger, Bacteroides, Roseburia, Bifidobacterium*, and *Akkermansia* were 5.84%, 6.35%, 6.65%, 4.47%, 11.09% and 1.99%, respectively.

**Table 1.** Baseline characteristics of 90 patients

Variable	Value
**Age, yr**	60(49–66.25)
**Male,n**	57 (63.33%)
**Body mass index, kg/m^2^**	25.56(22.65–28.67)
**Systolic blood pressure, mmHg**	127 ± 15
**Smoking status**	
Never	28(31.11%)
Past	30(33.33%)
Current	32(35.56%)
**Drinking status, standard drinks/wk**	
Never	65(72.22%)
1–4	10(11.11%)
5–8	11(12.22%)
>8	4(4.44%)
**Basic diseases**	
coronary heart disease	55(61.11%)
hypertension	44(48.89%)
diabetes	13(14.44%)
hyperuricemia	45(50%)
**Laboratory results**	
White blood cells, ×10^9^/L	6.86 ± 2.19
Neutrophilicgranulocyte,%	58.79 ± 12.89
Hemoglobin, ×10^12^/L	142(131–150.25)
Platelets, ×10^9^/L	216(174.75–258)
blood urea nitrogen,mmol/L	5.1(4.2–5.95)
Creatinine,umol/L	68.87(60–77)
uric acid,umol/L	389(306–438.1)
Glucose, mmol/L	4.86(4.32–5.77)
Total cholesterol, mmol/L	3.77(3.08–4.54)
Triglyceride, mmol/L	1.52(1.17–2.35)
high density lipoprotein,mmol/L	140.54 ± 18.14
total bilirubin,umol/L	11.3(8.6–14.2)
γ-glutamyl transpeptadase,U/L	25.5(18–39.25)

Data are presented as median (interquartile range), mean±SD, or number (%).

### 3.2 Analysis of the factors influencing the intestinal flora

Through cluster analysis, it was found that the participants enrolled in the study could be divided into two enterotypes according to different bacterial species compositions (see Figure 1(b-c)). The basic data and clinical indicators of participants with different enterotypes were compared and analyzed. (See Table 2). There were no significant differences in age, sex, BMI, or admission blood pressure between the two groups. The basic diseases of the two groups were significantly different except for AH (p < 0.001). There were no obvious abnormalities in the distribution of coronary heart disease, hypertension or diabetes. There was no significant difference in living habits for cohorts in two groups, such as the percentage of smoker and alcohol user. Laboratory test indicators were evaluated in each group, and the results suggested significant differences in blood uric acid levels in the two groups. According to the above results, it is speculated that uric acid levels might affect the structure of the intestinal flora.

**Table 2.** Clinical characteristics of patients according to enterotype

Variable	Enterotype 1 (n = 50)	Enterotype2 (n = 40)	P value
**Age, yr**	58.5(46.75–68.25)	62(52.25–65)	0.804
**Male,n**	33(66%)	24(60%)	0.356
**Body mass index, kg/m^2^**	25.2(20.43–28.55)	25.82(24.06–28.99)	0.162
**Systolic blood pressure, mmHg**	128 ± 14	125 ± 15	0.306
**Smoking status**			0.375
Never or Past	31(62%)	27(67.5%)	
Current	19(38%)	13(32.5%)	
**Drinking status**			0.388
Non-heavy drinker	35(70%)	30(75%)	
Heavy drinker*	15(30%)	10(25%)	
**Basic diseases**			
coronary heart disease	31(62%)	24(60%)	0.509
hypertension	26(52%)	18(45%)	0.222
diabetes	9(18%)	4(10%)	0.327
hyperuricemia	9(18%)	36(90%)	<0.001
**Laboratory results**			
White blood cells, ×10^9^/L	6.57 ± 1.88	7.03 ± 2.56	0.964
Neutrophilicgranulocyte,%	56.54 ± 12.62	60.78 ± 12.8	0.341
Hemoglobin, ×10^12^/L	142(125.5–151.25)	140(131–146)	0.586
Platelets, ×10^9^/L	216(174–263.5)	210(151.5–254.5)	0.145
blood urea nitrogen,mmol/L	4.6(4–6.03)	5.65(4.3–6.63)	0.045
Creatinine,umol/L	63(54–73.13)	71(60–78.75)	0.019
UA,umol/L	321.5(261–393.5)	441.35(412.25–494)	<0.001
Glucose, mmol/L	4.69(4.29–5.76)	4.89(4.56–5.61)	0.572
Total cholesterol, mmol/L	3.41(2.7–4.28)	4.09(3.3–4.93)	0.006
Triglyceride, mmol/L	1.46(1.09–2.38)	1.63(1.09–2.51)	0.15
high density lipoprotein,mmol/L	1.12 ± 0.28	1.13 ± 0.34	0.699
total bilirubin,umol/L	11.7(8.55–14.93)	11(8.73–13.99)	0.878
γ-glutamyl transpeptadase,U/L	21(16.75–43.63)	30.5(21.18–38.33)	0.273

Data are presented as median (interquartile range), mean±SD, or number (%).*Drinking status, more than 8 standard drinks/wk.

### 3.3 Asymptomatic Hyperuricemia affects the distribution of the intestinal flora

**Figure 3.**
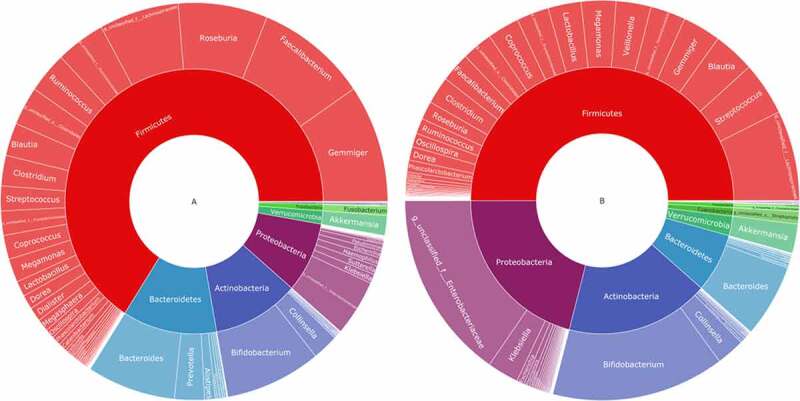
Taxonomic composition of the gut microbiome according to the asymptomatic hyperuricemia and control groups. double pie chart (inner circle: phylum level, outer circle: genus level). A: the asymptomatic hyperuricemia groups; B:control groups

To understand how AH could affect the intestinal flora, a double-layer pie chart was drawn for the patients with AH and the control group, as shown in (Figure 3(a-b)). In patients with AH, at the phylum level, the relative abundances of most abundant flora, including *Firmicutes, Actinobacteria, Bacteroidetes*, and *Proteobacteria* were 65.98%, 10.85%, 11.82%, and 8.59%, respectively; at the genus level, the relative abundances ofmost abundant flora, *including Faecalibacterium, Gemmiger, Bacteroides, Roseburia, Bifidobacterium*, and *Akkermansia* were 9.27%, 9.59%, 7.39%, 7.46%, 8.01% and 1.61%, respectively. In the patients from the control group, at the phylum level, the relative abundances of *Firmicutes, Actinobacteria, Bacteroidetes*, and *Proteobacteria* were 50.03%, 17.62%, 7.15%, and 20.94%, respectively; at the genus level, the relative abundances of *Faecalibacterium, Gemmiger, Bacteroides, Roseburia, Bifidobacterium*, and *Akkermansia* were 2.41%, 3.12%, 5.92%, 1.48%, 14.18% and 2.36%, respectively.

**Figure 4.**
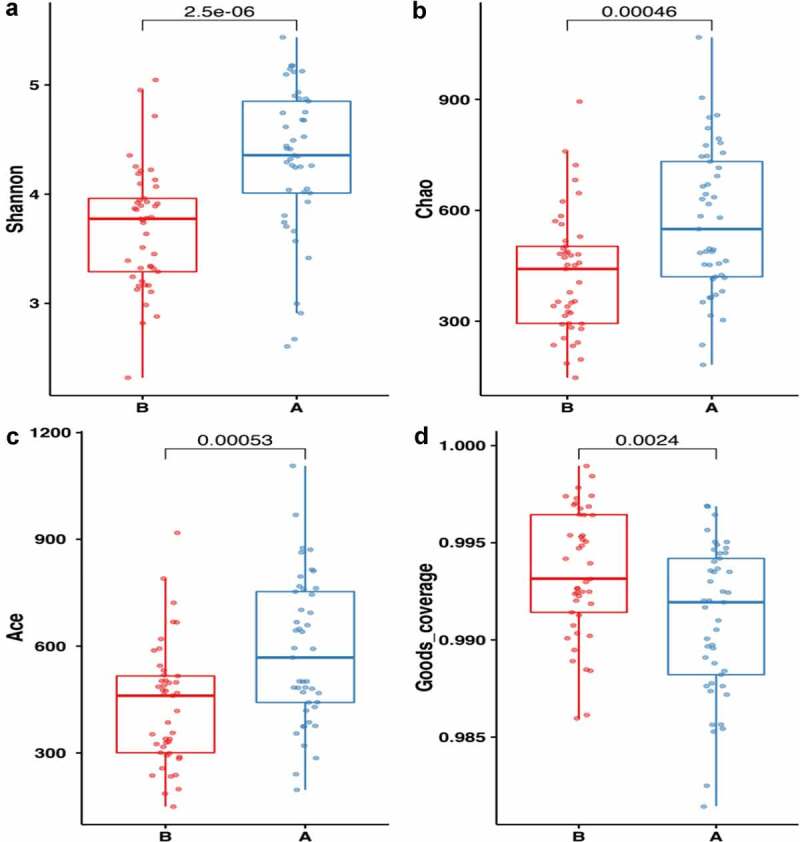
Comparison of fecal microbial diversity, as estimated by the Shannon index(a), Chao1 index(b), Ace index(c) and goods_coverage index(d). A: the asymptomatic hyperuricemia groups; B:control groups

**Figure 5.**
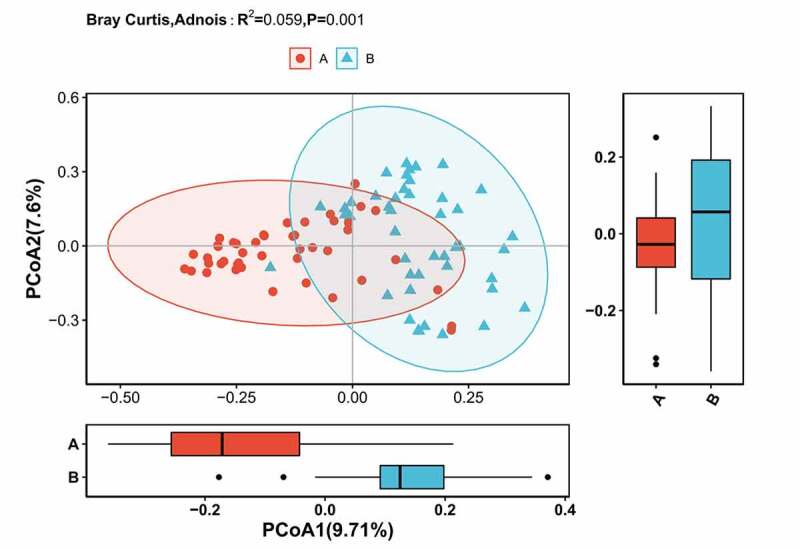
Differences in beta diversity indices between asymptomatic hyperuricemia and healthy groups measured with Adonis test and PCOA. The horizontal and vertical axes represent the first and second principal coordinates explaining the greatest proportion of variance to the bacterial communities (showed by percentage). A: the asymptomatic hyperuricemia groups; B:control groups; PCOA: principal coordinates analysis

Subsequently, alpha diversity analysis of the two groups was performed (see Figure 4(a-d)). The results revealed higher Chao1 and Ace index assessments in the AH group, indicating that the abundance of operational taxonomic units (OTUs) in the AH group was greater than that in the control group. Shannon results also revealed higher community diversity in the AH group than in the control group. The Good’s coverage index was close to 1 between the two groups, and the control group was higher, suggesting that the sequencing depth was reasonable. Namely, this sequencing depth had basically covered all the species in the sample. Furthermore, it was more apparent in the control group. The Bray-Curtis distance was used for beta diversity analysis, and the results are displayed in the form of principal coordinate analysis (PCOA), as shown in Figure 5. In addition, we conducted an Adnois analysis on the intragroup differences, and the results showed R2 = 0.059 and P = 0.001. All the species diversity analyses suggested significant differences between the two groups. These results indicated changes in the composition of the intestinal microbiome during the occurrence and development of AH.

### 3.4 Potential Microbiota Biomarkers of Asymptomatic Hyperuricemia

**Figure 6.**
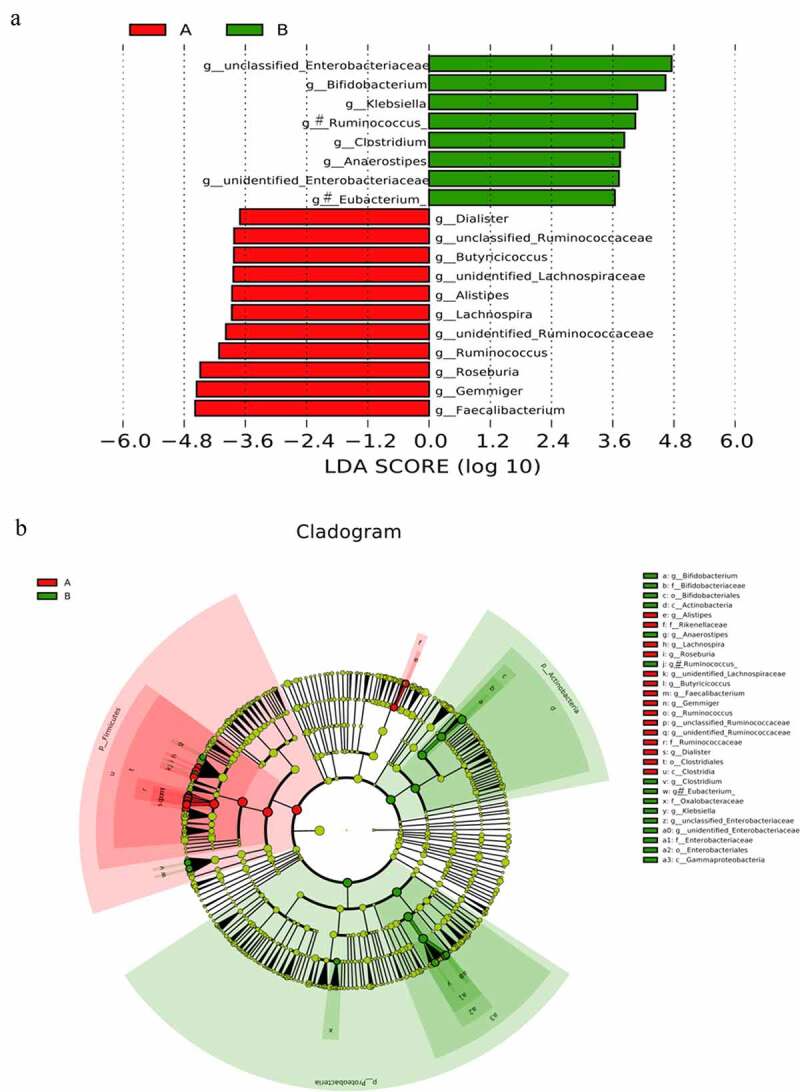
(a) clustering tree showing significant taxonomic differences in the gut microbiota between AH (negative score) and control (positive score) groups through LEfSe analysis (LDA scores (log10) >3). (b) Cladogram indicating the phylogenetic distribution of the gut microbiota in AH and control groups through LEfSe analysis (LDA scores (log10) >3). AH: asymptomatic hyperuricemia; p: phylum; c: class; o: order; f: family; g: genus; LEfSe: linear discriminant analysis effect size; LDA: linear discriminant analysis. #:Tentative names in greenenes database

**Figure 7.**
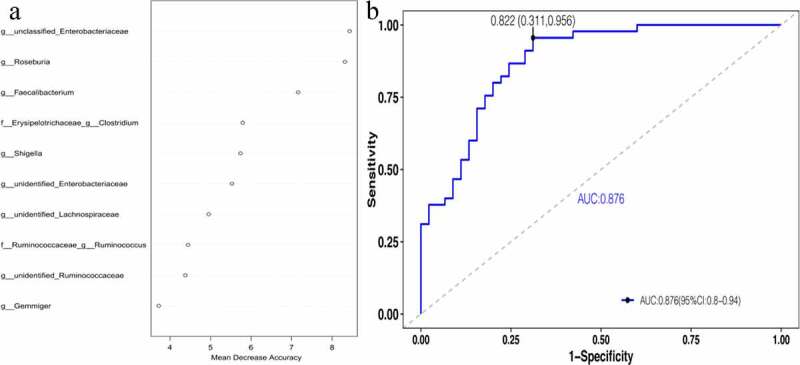
Important biomarkers.[A] The top 10 bacteria belong to the genus level.[B] ROCs curve with AUC for the diagnostic performance of the gut microbial model

To explore potential biomarkers, the linear discriminant analysis effect size was used to identify species that differed significantly between the two groups. Among them, the linear discriminant analysis threshold is larger than 3. The results indicated that there were 19 types of bacteria with different levels of bacterial genera between the two groups. The bacteria enriched in the AH group included the following: *unclassified_Ruminococcaceae, Alistipes, Dialister, unidentified_Ruminococcaceae, Roseburia, Gemmiger*, and *Faecalibacterium*. The bacteria included in the control group were as follows: *unclassified_Enterobacteriaceae, Bifidobacterium, Klebsiella, [Ruminococcus], unidentified_Lactobacill-ales, unclassified_Enterococcaceae, [Eubacterium], unidentified_Enterobacteriaceae*, and *Clostridium*. Simultaneously, a branching diagrams for the difference of bacteria between two groups was drawn (see Figure 6(a-b)). Subsequently, the random forest model was used to identify the top 10 bacteria that contributed the most discrimination between the two groups. The results are shown in (Figure 7(a)). There were many overlaps between the bacteria found through random forest and LEFSE analysis, and the results suggested that these bacteria might be the key bacteria that affected the occurrence and development of AH. Finally, 10 bacteria searched by random forest were jointly diagnosed for AH, and the ROC curve was plotted. The results are shown in (Figure 7(b)). From the figure, we could see that the diagnostic area AUC value of 10 bacteria (such as *unclassified_Enterobacteriaceae*) for AH was 0.876. This result revealed that this diagnostic model was good, and this type of bacteria was closely related to AH.

## 4. Discussion

In this study, the phylum-level bacteria *Firmicutes* and *Bacteroidetes* accounted for approximately 68% of the overall population, and the phylum-level bacteria *Proteobacteria* and *Actinobacteria* accounted for 14.8% and 14.2%, respectively. The phylum *Firmicutes* and the phylum *Bacteroidetes* belong to the dominant flora in humans. In this study, *Firmicutes* and *Bacteroidetes* were generally less abundant. Considering that most of the samples we included were middle-aged and elderly individuals with chronic diseases, we hold that this sample structure is more in line with the existing increasingly aging social structure. Subsequently, the two groups were compared by intestinal cluster analysis, and the results indicated that AH might be associated with gut flora. This conclusion was consistent with existing studies, and this approach was adopted by many scholars in the past [16,17]. In species composition-based clustering analysis and previous studies, we further focused on the relationship between AH and intestinal flora. Through further bioinformatics dimensionality reduction analysis between the AH group and the control group, it was clear that there were significant differences in the gut flora structure between the two groups (Adnois analysis suggested that R^2^ = 0.059 and P = 0.001). Subsequently, we analyzed which bacteria had abundance differences between the AH and control groups and explored which bacteria were the most important between the two groups. The top 10 most important bacteria were incorporated into the intestinal flora to establish a diagnostic model for AH. The AUC area showed that the diagnostic model had a good diagnostic effect, which provided ideas for the further study of probiotics. In this study, *Alistipes* bacteria were highly abundant in patients with AH. and the genus *Alistipes* was likely to be associated with purine metabolism. Recent studies on this bacterium have reported that the abundance of *Alipipes* bacteria is high in the smoke carcinogenic model, and purine metabolism is also abnormal at the metabolic level of the model. Simultaneously, the genus *Alipipes* seems to be related to the occurrence and development of nephrotic diseases. Abnormal abundance of *Alipipes* was detected in different models of renal obstruction or nephropathy. Combined with the results of this study and previous studies, *Alistipes* may be related to purine metabolism, suggesting that this bacterium may aggravate AH and develop into hyperuricaemic nephropathy. The genus *Dialister* and the genus *Alipipes* are both reported for the first time in studies such as AH. In previous intestinal microbiological studies, the abundance of most genera of *Dialister* increased in different disease groups [18,19]. This result also revealed intestinal imbalance in AH. In previous studies, the relationship between the genus *Roseburia* and AH has been reported. In 2020, some researchers have made detailed reports on hyperuricemia nephropathy and intestinal flora. In a study, the abundance of the genus *Roseburia* was reduced in hyperuricemia nephropathy [20]. The genus *Roseburia* is a short-chain fatty acid-producing bacterium that can reduce inflammation levels. In combination with the existing research considerations, *Roseburia* is likely to be a compensatory increase in the prehyperuricaemia stage. In the later stages of the disease, such as in the stage of hyperuricemia nephropathy, there is decompensation, and the abundance of the flora decreases. There are many reports on the dynamic changes of intestinal microbes in the occurrence and development of diseases, which also provides the possibility for probiotics to treat diseases. For the genus *Gemmiger*, the abundance of AH was relatively high, which is consistent with the results of existing studies. Recent studies have reported that the genus *Gemmiger* was mentioned in research on the influence of Hericium erinaceus on the intestinal flora. In a study, it was found that this genus was correlated with clinical indicators such as uric acid in patients [21]. This result is similar to the results of our study, suggesting that *Gemmiger* is related to uric acid levels. Similar to the genus *Roseburia*, the genus *Faecalibacterium* is also a short-chain fatty acid-producing bacterium. At present, research on these bacteria is relatively mature in intestinal diseases such as colitis, and it has been experimentally confirmed that there is a probiotic effect in intestinal disease models [22]. With more mature research in the future, this bacterium is expected to expand its indications and become a new generation of probiotics. The relationship between the genus *Faecalibacterium* and gout has been reported and is similar to that of the genus *Roseburia*. In the existing reports, the genus *Faecalibacterium* has low abundance in the disease group, which is contrary to the results of our study [23]. According to the characteristics of the population in this study and previous research reports on this bacterium, it is considered that the increase in this bacterium in the AH population is related to early disease. This increase in bacterial abundance should be a compensatory increase and should play an anti-inflammatory role in the early stage of the disease. The dynamic change of the flora should be a common phenomenon among many floras, and these bacteria with obvious fluctuation should be the research direction of probiotics. In this study, in the AH population, the abundance of unidentified and unclassified bacteria in *Ruminococcaceae* bacteria was relatively high. It accounts for a higher importance in the random model. Past studies on *Ruminococcaceae* bacteria have reported that this bacterium is closely related to gout and uric acid metabolism, which is similar to the conclusions of this study [24].

In addition to reporting the high-abundance bacteria in the population of the AH population, this study also reported the composition of bacteria with low gut microbiota in AH. The genus *Bifidobacterium* is generally recognized as a probiotic that has many significant physiological functions, such as acting as a biological barrier for human health, enhancing immunity [25], fighting against tumors [26], improving gastrointestinal function, and resisting aging [27]. The genus *Bifidobacterium* can inhibit the growth of harmful bacteria, resist pathogen infection, synthesize vitamins needed by the human body, promote the absorption of minerals, produce acetic acid, acetic acid propiononic acid, butyric acid and lactic acid to stimulate intestinal peristalsis, promote defecation, prevent constipation and inhibit intestinal corruption, purify the intestinal environment, decompose carcinogens, and stimulate the human immune system to improve disease resistance. In this study, the abundance of the genus *Bifidobacterium* in the population of the AH was very low, and this result is consistent with previous studies [28]. This result suggests that there is flora imbalance in the AH population. In 2020, some scholars have made detailed reports on the relationship between *Klebsiella* and uric acid. Researchers verified that *Klebsiella* is closely related to uric acid levels through experiments such as bacterial infection of macrophages and suggested that the genus *Klebsiella* functions by regulating autophagy and inflammation. This result is consistent with the conclusion of this study [29]. The low abundance of the genus *Clostridium* in the population of the AH has also been reported by previous studies [30]. Currently, there are many research reports on the genus *Clostridium*. In China, some researchers have applied *Clostridium* to carry out animal experiments on hyperuricemia model mice, which proved that this bacteria had a certain improvement effect on uric acid levels [21]. In combination with the existing studies, we consider the genus *Clostridium* with great potential to become a new generation of probiotics. However, this genus has a large family with many subordinate species-level bacteria. Except for a few pathogenic bacteria, most of them are nonpathogenic bacteria, whose effect has not been clear thus far. Therefore, it is of great significance to select and screen *Clostridium* and verify animal experiments to reduce the beneficial effects of uric acid. This genus was also mentioned in a randomized forest diagnostic model of this study, suggesting that this bacterium is likely to have the potential value of probiotics. In this study, the abundance of the genus *[Ruminococcus]* and the genus *[Eubacterium]* in the population of the AH was very low. At present, the genus *Ruminococcus* and the genus *Eubacterium* are mentioned in the population of AH and the population of gout populations, which are similar to the results of this study [8]. In this study, the reduced bacteria in the AH population also had unidentified and unclassified bacteria with low abundance, such as *unclassified_Enterobacteriaceae, unidentified_Ente-robacteriaceae, unidentified*_*Lactobacillales*, and *unclassified_Enterococcaceae*. Reports on the relationship between *Enterobacteriaceae, Lactobacilla-les*, and gout and other related diseases are similar to the results of this study [31]. The genus *Enterococcaceae* seems to be closely related to purine metabolism in existing studies, and this phenomenon is consistent with the results of this study.

This study is the first to systematically report the population of AH by using microbial informatics. AH is the early stage of gout. Systematic reporting of this population is very important to explore the study of intestinal flora and gout. In addition to reporting that there are significantly different bacteria between the two groups, this is also the first study to explore the bacteria that play an important role in the two groups in AH by means of random forest and other mechanical learning models and verify that the important bacteria screened by this method are closely related to the disease by using the idea of a joint diagnosis model. Combined with the results of this study and the above discussion, it is found that patients with AH had intestinal flora imbalance. In this flora imbalance, there are not only bacteria with higher *Alistipes* but also probiotics with higher bacteria, such as *Roseburia* and *Faecalibacterium*. The increase in such probiotics is speculated to be a compensatory phenomenon. The results of this study provide an important basis for exploring the treatment of gout with probiotics.

## 5. Limitations

There are limitations to the current study. In this study, there were some unidentified and unclassified bacteria and tentatively named bacteria, such as *[Ruminococcus]* bacteria and *[Eubacterium]* bacteria. Considering that it is related to the accuracy of 16S rRNA sequencing, we consider this is also one of the insufficiencies in this study. In addition, the sample size included in this study was small, resulting in failure to further verify the stability of the diagnostic model. It is also the insufficiency of this study. Finally, as a cross-sectional study, the results are not enough to explain the causal relationship between ah and intestinal biota. In summary, larger longitudinal studies focusing on AH are required to confirm our results.

## 6. Conclusion

The AH affects the intestinal flora. In the AH population, the abundance of bacteria such as *Alipipes, Dialister, Roseburia, Gemmiger*, and *Faecalibacterium* was relatively high, while the abundance of bacteria such as *Bifidobacterium, Klebsiella*, and *Clostridium* was relatively low. In combination with previous studies, *Roseburia* and *Faecalibacterium* may have a modern compensatory increase in the early stage of the disease during the continuous aggravation of HUA, and the decompensation of these bacteria is likely to decrease in the later stage of the disease. Diagnostic models have confirmed that bacteria, such as *unclassified Enterobacteriaceae, Roseburia*, and *Faecalibacte-rium*, can have good diagnostic value for AH.

## Highlights

● The intestinal flora of asymptomatic hyperuricemia patients was significantly different from that of healthy people.

● The abundance of intestinal bacteria in the asymptomatic hyperuricemia population was higher than that in the healthy population.

● The abundance of some probiotics in the intestinal flora of the asymptomatic hyperuricemia population is high.
